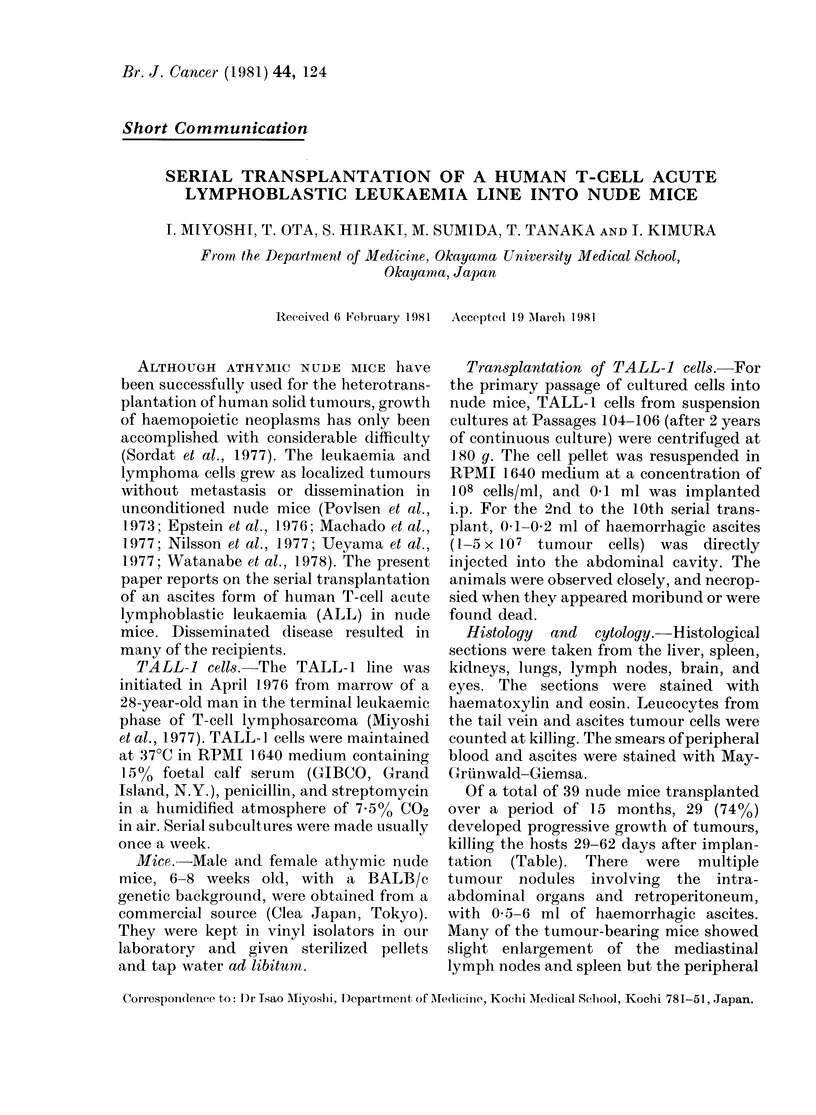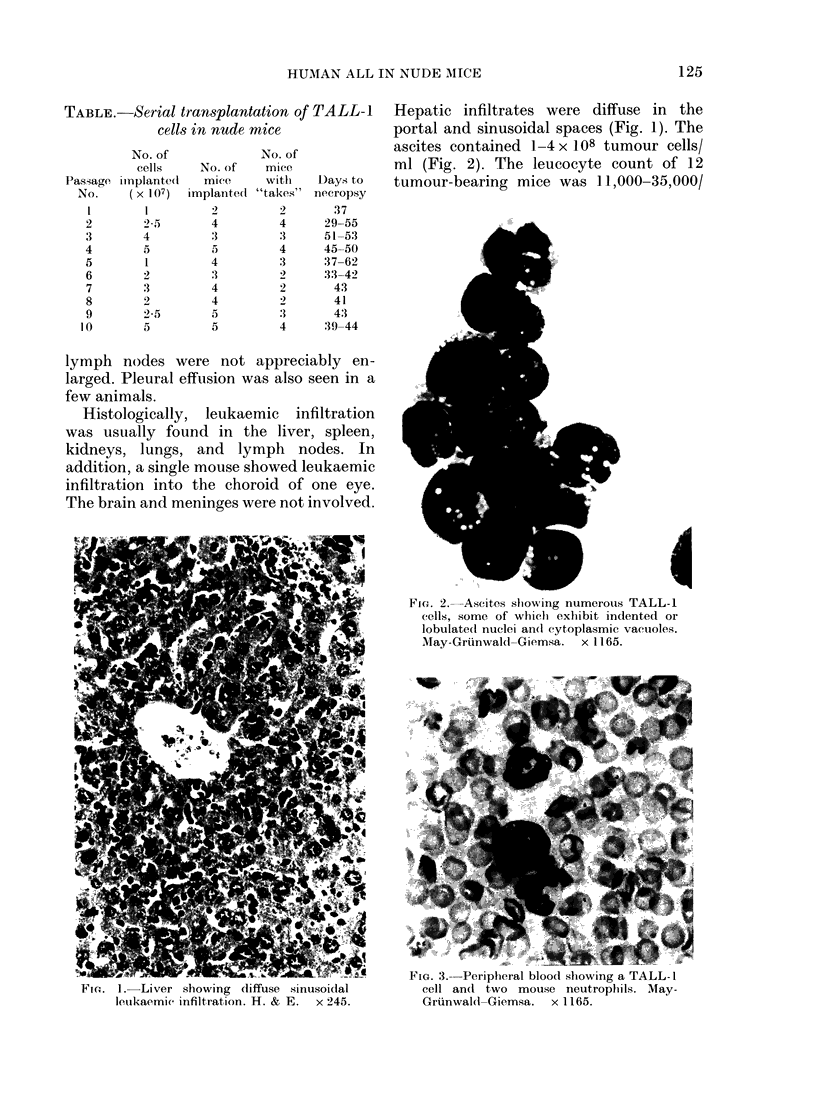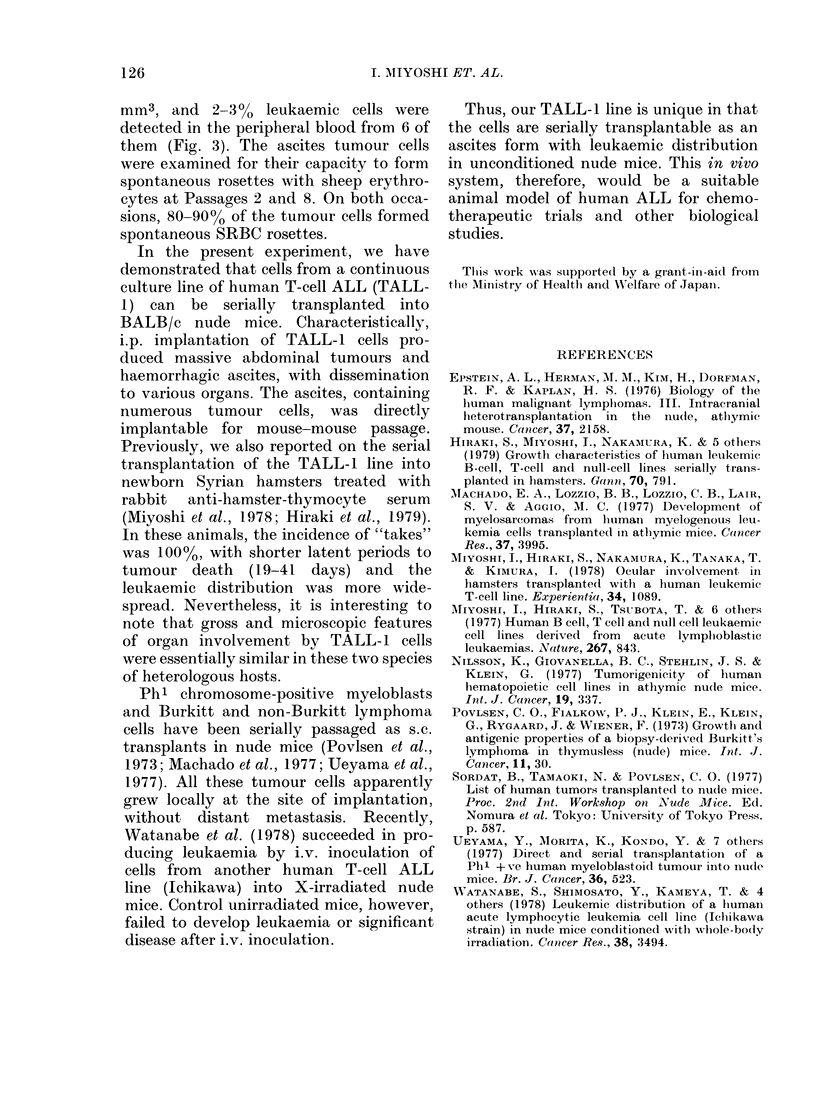# Serial transplantation of a human T-cell acute lymphoblastic leukaemia line into nude mice.

**DOI:** 10.1038/bjc.1981.157

**Published:** 1981-07

**Authors:** I. Miyoshi, T. Ota, S. Hiraki, M. Sumida, T. Tanaka, I. Kimura

## Abstract

**Images:**


*Br. J. Cancer* (1981) **44,** 124

***Short Communication***

### SERIAL TRANSPLANTATION OF A HUMAN T-CELL ACUTE LYMPHOBLASTIC LEUKAEMIA LINE INTO NUDE MICE

I. MIYOSHI, T. OTA, S. HIRAKI, M. SUMIDA, T. TANAKA AND I. KIMURA

*From the Department of Medicine, Okayama University Medical School, Okayama, Japan*

Received 6 February 1981 Accepted 19 March 1981

ALTHOUGH ATHYMIC NUDE MICE have been successfully used for the heterotransplantation of human solid tumours, growth of haemopoietic neoplasms has only been accomplished with considerable difficulty (Sordat *et al.*, 1977). The leukaemia and lymphoma cells grew as localized tumours without metastasis or dissemination in unconditioned nude mice (Povlsen *et al.*, 1973; Epstein *et al.*, 1976; Machado *et al.*, 1977; Nilsson *et al.*, 1977; Ueyama *et al.*, 1977; Watanabe *et al.*, 1978). The present paper reports on the serial transplantation of an ascites form of human T-cell acute lymphoblastic leukaemia (ALL) in nude mice. Disseminated disease resulted in many of the recipients.

*TALL-1 cells.*—The TALL-1 line was initiated in April 1976 from marrow of a 28-year-old man in the terminal leukaemic phase of T-cell lymphosarcoma (Miyoshi *et al.*, 1977). TALL-1 cells were maintained at 37°C in RPMI 1640 medium containing 15% foetal calf serum (GIBCO, Grand Island, N.Y.), penicillin, and streptomycin in a humidified atmosphere of 7·5% $CO_2$ in air. Serial subcultures were made usually once a week.

*Mice.*—Male and female athymic nude mice, 6–8 weeks old, with a BALB/c genetic background, were obtained from a commercial source (Clea Japan, Tokyo). They were kept in vinyl isolators in our laboratory and given sterilized pellets and tap water *ad libitum.*

*Transplantation of TALL-1 cells.*—For the primary passage of cultured cells into nude mice, TALL-1 cells from suspension cultures at Passages 104–106 (after 2 years of continuous culture) were centrifuged at 180 *g*. The cell pellet was resuspended in RPMI 1640 medium at a concentration of $10^8$ cells/ml, and 0·1 ml was implanted i.p. For the 2nd to the 10th serial transplant, 0·1–0·2 ml of haemorrhagic ascites ($1–5 \times 10^7$ tumour cells) was directly injected into the abdominal cavity. The animals were observed closely, and necropsied when they appeared moribund or were found dead.

*Histology and cytology.*—Histological sections were taken from the liver, spleen, kidneys, lungs, lymph nodes, brain, and eyes. The sections were stained with haematoxylin and eosin. Leucocytes from the tail vein and ascites tumour cells were counted at killing. The smears of peripheral blood and ascites were stained with May-Grünwald–Giemsa.

Of a total of 39 nude mice transplanted over a period of 15 months, 29 (74%) developed progressive growth of tumours, killing the hosts 29–62 days after implantation (Table). There were multiple tumour nodules involving the intra-abdominal organs and retroperitoneum, with 0·5–6 ml of haemorrhagic ascites. Many of the tumour-bearing mice showed slight enlargement of the mediastinal lymph nodes and spleen but the peripheral

Correspondence to: Dr Isao Miyoshi, Department of Medicine, Kochi Medical School, Kochi 781-51, Japan.

TABLE.—*Serial transplantation of TALL-1 cells in nude mice*

| Passage No. | No. of cells implanted (× 10⁷) | No. of mice implanted | No. of mice with "takes" | Days to necropsy |
|---|---|---|---|---|
| 1 | 1 | 2 | 2 | 37 |
| 2 | 2·5 | 4 | 4 | 29–55 |
| 3 | 4 | 3 | 3 | 51–53 |
| 4 | 5 | 5 | 4 | 45–50 |
| 5 | 1 | 4 | 3 | 37–62 |
| 6 | 2 | 3 | 2 | 33–42 |
| 7 | 3 | 4 | 2 | 43 |
| 8 | 2 | 4 | 2 | 41 |
| 9 | 2·5 | 5 | 3 | 43 |
| 10 | 5 | 5 | 4 | 39–44 |

lymph nodes were not appreciably enlarged. Pleural effusion was also seen in a few animals.

Histologically, leukaemic infiltration was usually found in the liver, spleen, kidneys, lungs, and lymph nodes. In addition, a single mouse showed leukaemic infiltration into the choroid of one eye. The brain and meninges were not involved.

FIG. 1.—Liver showing diffuse sinusoidal leukaemic infiltration. H. & E. × 245.

Hepatic infiltrates were diffuse in the portal and sinusoidal spaces (Fig. 1). The ascites contained $1–4 \times 10^8$ tumour cells/ml (Fig. 2). The leucocyte count of 12 tumour-bearing mice was 11,000–35,000/

FIG. 2.—Ascites showing numerous TALL-1 cells, some of which exhibit indented or lobulated nuclei and cytoplasmic vacuoles. May-Grünwald–Giemsa. × 1165.

FIG. 3.—Peripheral blood showing a TALL-1 cell and two mouse neutrophils. May-Grünwald–Giemsa. × 1165.

mm$^3$, and 2–3% leukaemic cells were detected in the peripheral blood from 6 of them (Fig. 3). The ascites tumour cells were examined for their capacity to form spontaneous rosettes with sheep erythrocytes at Passages 2 and 8. On both occasions, 80–90% of the tumour cells formed spontaneous SRBC rosettes.

In the present experiment, we have demonstrated that cells from a continuous culture line of human T-cell ALL (TALL-1) can be serially transplanted into BALB/c nude mice. Characteristically, i.p. implantation of TALL-1 cells produced massive abdominal tumours and haemorrhagic ascites, with dissemination to various organs. The ascites, containing numerous tumour cells, was directly implantable for mouse–mouse passage. Previously, we also reported on the serial transplantation of the TALL-1 line into newborn Syrian hamsters treated with rabbit anti-hamster-thymocyte serum (Miyoshi *et al.*, 1978; Hiraki *et al.*, 1979). In these animals, the incidence of "takes" was 100%, with shorter latent periods to tumour death (19–41 days) and the leukaemic distribution was more widespread. Nevertheless, it is interesting to note that gross and microscopic features of organ involvement by TALL-1 cells were essentially similar in these two species of heterologous hosts.

Ph$^1$ chromosome-positive myeloblasts and Burkitt and non-Burkitt lymphoma cells have been serially passaged as s.c. transplants in nude mice (Povlsen *et al.*, 1973; Machado *et al.*, 1977; Ueyama *et al.*, 1977). All these tumour cells apparently grew locally at the site of implantation, without distant metastasis. Recently, Watanabe *et al.* (1978) succeeded in producing leukaemia by i.v. inoculation of cells from another human T-cell ALL line (Ichikawa) into X-irradiated nude mice. Control unirradiated mice, however, failed to develop leukaemia or significant disease after i.v. inoculation.

Thus, our TALL-1 line is unique in that the cells are serially transplantable as an ascites form with leukaemic distribution in unconditioned nude mice. This *in vivo* system, therefore, would be a suitable animal model of human ALL for chemotherapeutic trials and other biological studies.

This work was supported by a grant-in-aid from the Ministry of Health and Welfare of Japan.

### REFERENCES

Epstein, A. L., Herman, M. M., Kim, H., Dorfman, R. F. & Kaplan, H. S. (1976) Biology of the human malignant lymphomas. III. Intracranial heterotransplantation in the nude, athymic mouse. *Cancer*, **37**, 2158.

Hiraki, S., Miyoshi, I., Nakamura, K. & 5 others (1979) Growth characteristics of human leukemic B-cell, T-cell and null-cell lines serially transplanted in hamsters. *Gann*, **70**, 791.

Machado, E. A., Lozzio, B. B., Lozzio, C. B., Lair, S. V. & Aggio, M. C. (1977) Development of myelosarcomas from human myelogenous leukemia cells transplanted in athymic mice. *Cancer Res.*, **37**, 3995.

Miyoshi, I., Hiraki, S., Nakamura, K., Tanaka, T. & Kimura, I. (1978) Ocular involvement in hamsters transplanted with a human leukemic T-cell line. *Experientia*, **34**, 1089.

Miyoshi, I., Hiraki, S., Tsubota, T. & 6 others (1977) Human B cell, T cell and null cell leukaemic cell lines derived from acute lymphoblastic leukaemias. *Nature*, **267**, 843.

Nilsson, K., Giovanella, B. C., Stehlin, J. S. & Klein, G. (1977) Tumorigenicity of human hematopoietic cell lines in athymic nude mice. *Int. J. Cancer*, **19**, 337.

Povlsen, C. O., Fialkow, P. J., Klein, E., Klein, G., Rygaard, J. & Wiener, F. (1973) Growth and antigenic properties of a biopsy-derived Burkitt's lymphoma in thymusless (nude) mice. *Int. J. Cancer*, **11**, 30.

Sordat, B., Tamaoki, N. & Povlsen, C. O. (1977) List of human tumors transplanted to nude mice. *Proc. 2nd Int. Workshop on Nude Mice*. Ed. Nomura *et al.* Tokyo: University of Tokyo Press. p. 587.

Ueyama, Y., Morita, K., Kondo, Y. & 7 others (1977) Direct and serial transplantation of a Ph$^1$ +ve human myeloblastoid tumour into nude mice. *Br. J. Cancer*, **36**, 523.

Watanabe, S., Shimosato, Y., Kameya, T. & 4 others (1978) Leukemic distribution of a human acute lymphocytic leukemia cell line (Ichikawa strain) in nude mice conditioned with whole-body irradiation. *Cancer Res.*, **38**, 3494.